# scRNA-seq characterizing the heterogeneity of fibroblasts in breast cancer reveals a novel subtype SFRP4^+^ CAF that inhibits migration and predicts prognosis

**DOI:** 10.3389/fonc.2024.1348299

**Published:** 2024-04-15

**Authors:** Lvwen Ning, Chuntao Quan, Yue Wang, Zhijie Wu, Peixiu Yuan, Ni Xie

**Affiliations:** ^1^ Biobank, Shenzhen Second People’s Hospital, First Affiliated Hospital of Shenzhen University, Health Science Center, Shenzhen University, Shenzhen, China; ^2^ Shenzhen Institute of Advanced Technology, Chinese Academy of Sciences, Shenzhen, China; ^3^ College of Materials and Energy, South China Agricultural University, Guangzhou, China

**Keywords:** breast cancer, cancer-associated fibroblast, SFRP4+ CAF, prognosis, heterogeneity, WNT, migration

## Abstract

**Introduction:**

Cancer-associated fibroblasts (CAFs) are a diverse group of cells that significantly impact the tumor microenvironment and therapeutic responses in breast cancer (BC). Despite their importance, the comprehensive profile of CAFs in BC remains to be fully elucidated.

**Methods:**

To address this gap, we utilized single-cell RNA sequencing (scRNA-seq) to delineate the CAF landscape within 14 BC normal-tumor paired samples. We further corroborated our findings by analyzing several public datasets, thereby validating the newly identified CAF subtype. Additionally, we conducted coculture experiments with BC cells to assess the functional implications of this CAF subtype.

**Results:**

Our scRNA-seq analysis unveiled eight distinct CAF subtypes across five tumor and six adjacent normal tissue samples. Notably, we discovered a novel subtype, designated as SFRP4+ CAFs, which was predominantly observed in normal tissues. The presence of SFRP4+ CAFs was substantiated by two independent scRNA-seq datasets and a spatial transcriptomics dataset. Functionally, SFRP4+ CAFs were found to impede BC cell migration and the epithelial-mesenchymal transition (EMT) process by secreting SFRP4, thereby modulating the WNT signaling pathway. Furthermore, we established that elevated expression levels of SFRP4+ CAF markers correlate with improved survival outcomes in BC patients, yet paradoxically, they predict a diminished response to neoadjuvant chemotherapy in cases of triple-negative breast cancer.

**Conclusion:**

This investigation sheds light on the heterogeneity of CAFs in BC and introduces a novel SFRP4+ CAF subtype that hinders BC cell migration. This discovery holds promise as a potential biomarker for refined prognostic assessment and therapeutic intervention in BC.

## 1 Introduction

Breast cancer (BC) is a heterogeneous disease that involves interactions between the malignant cells and the various types of stromal cells in the tumor microenvironment (1); among these stromal cells, cancer-associated fibroblasts (CAFs) are the most abundant and have been shown to play important roles in tumor initiation, progression, and response to therapy (2–5). CAFs produce the most important components of the extracellular matrix (ECM) to physically interfere with cancer cells (6). CAFs also generate matrix-crosslinking enzymes to mediated ECM remodeling, thereby increasing tumor stiffness (7). In addition, CAFs also secrete multiple growth factors, cytokines and exosomes which promote tumor growth and modulate therapy responses (8–11). These CAF-derived factors can also act on a range of immune cells to cause immunosuppressive and immunopromoting consequences (12–14). Öhlund et al. classified CAFs into two categories, myCAF or iCAF: myCAF has a matrix-producing contractile phenotype and iCAF is for regulation of inflammation (15). Subsequently, antigen-presenting CAF (apCAF), which expresses MHC class II and CD4 to activate CD4+ T cell, was reported (16). However, due to the limited marker availability and the intricate tumor microenvironment, the origin, diversity, and function of CAFs may not be fully characterized in BC (17, 18).

Single-cell RNA sequencing (scRNA-seq) is a powerful technique that can reveal the transcriptomic profiles and phenotypic heterogeneity of individual cells of a population (19). Since CAFs lack specific markers, one strategy is to use negative selection that isolates EpCAM−/CD45−/CD31−/NG2− cell to devoid epithelial cells, immune cells, endothelial cells, and pericytes. By applying this strategy, four CAF subsets, referred to as CAF-S1 to S4, were defined in human BC, based on the expression of six markers, including FAP, smooth-muscle α actin (SMA), integrin β1 (CD29), S100-A4/FSP1, PDGFRβ, and CAV1 (12, 20, 21). Subsequentially, Kieffer et al. further identified eight subclusters from FAP+ CAF-S1 (22). However, since this strategy enriched CAF cells in the first place, it may also miss some cells. Therefore, the other approach is to sequence all cells, and then annotate cells based on the expression pattern including CAFs. Using scRNA-seq, several studies have identified numerous distinct subpopulations of CAFs with different spatial distributions, molecular signatures, and functional properties. In an MMTV-PyMT BC mouse model, Bartoschek et al. defined four distinct CAF subpopulations attributed to different origins: vascular CAFs, matrix CAFs, cycling CAFs, and developmental CAFs (23). Moreover, Wu et al. defined two CAF subclusters with features of myofibroblasts (myCAFs) and inflammatory CAFs (iCAFs) with high expression of growth factors and immunomodulatory molecules in triple-negative breast cancer (TNBC) (24). Furthermore, Friedman et al. found that the expression profiles of CAF subpopulations changed from an immunoregulatory program to wound healing and antigen presentation programs during BC progression in mice (25). A single-cell atlas study in BC identified CAFs from other cell types using markers PDGFRA and COL1A1 and clustered them into five subgroups, differentiated by some marker genes: ALDH1A1, KLF4, LEPR, CXCL12, C3, ACTA2 (αSMA), TAGLN, FAP and COL1A1 (26).

However, the heterogeneity of CAF in BC is still under-characterized, especially since the current study focused on tumor samples. So, we performed scRNA-seq on seven paired BC and adjacent normal samples without prior selection of cells, to capture the changes from normal to tumor. This study reveals the cellular heterogeneity of CAF at single-cell resolution to help understand their roles in the occurrence and development of BC. We identified eight CAF subtypes that demonstrate different expression patterns, in which a new CAF subtype that specifically expresses the secreted frizzled-related protein 4 (SFRP4) and has a significantly higher composition in normal tissue, may inhibit BC progression by inhibiting the WNT signaling pathway.

## 2 Materials and methods

### 2.1 Clinical samples

The matched primary and adjacent normal samples were collected from seven BC patients during surgery and immediately stored in the GEXSCOPE Tissue Preservation Solution at 2-8°C. Histological characterization was determined according to the criteria of the World Health Organization by pathologists from the hospital. Pathologic staging was performed according to the current International Union against Cancer tumor–lymph node metastasis classification. Experiments were reviewed and approved by the Institutional Review Board of The Second Hospital of Shenzhen, China, and were conducted in compliance with the Helsinki Declaration. Each patient provided written informed consent before sample collection.

### 2.2 Single-cell RNA sequencing

Single-cell suspensions were at once processed for the scRNA-seq using the Chromium platform (10x Genomics). Single-cell capture, barcoding, and library preparation were performed by following the 10x Genomics Single Cell Chromium 3′ protocols, and the final libraries were sequenced on the Illumina HiSeq 2500 platform. Data was processed using the CASAVA pipeline (Illumina), and sequencing reads were converted to FASTQ files and UMI read counts using the CellRanger software (10x Genomics, v.2.1.1).

### 2.3 Single-cell RNA sequencing analysis

The single-cell sequencing reads from the 10x Genomics Chromium were demultiplexed and then aligned to the GRCh38.p12 human genome reference using the CellRanger pipeline (v.3.1.0, 10x Genomics) with the default parameters and UMI count matrices against the corresponding Ensembl gene annotations were generated. The reference genome and the gene annotation were downloaded from the UCSC genome browser (27).

Count matrices were further analyzed using Scanpy (https://github.com/scverse/scanpy) (version 1.9.3) (28). Of the fourteen samples, sample 5 and sample 9 were removed because limited cells were detected and sample 4 was removed because the expressed genes were abnormally lower than other samples. The count matrices of the remaining eleven samples were merged. Cells with fewer than 200 genes expressed were filtered and genes detected in less than 3 cells were filtered. In addition, cells with higher than 20% mitochondrial or 50% ribosomal RNAs were also filtered as they represented low-quality cells. Further, for potential doublets, we did not include cells expressing more than 5000 genes and used scDblFinder (29) to further mark the remaining cells. We also calculated the cell cycle scores for each cell using *sc.tl.score_genes_cell_cycle*.

Then the matrix was normalized based on total UMI counts per cell (1e4) and then log-transformed. To reduce potential batch effects, a graph-based data integration algorithm batch-balanced KNN (bbknn) (30) was applied to the matrix. Ridge regression (31) was also performed on samples and Leiden clusters. The integrated data was then used for downstream analysis. Highly variable genes were identified using *sc.pp.highly_variable_genes* from scanpy with parameters min_mean=0.0125, max_mean=3, min_disp=0.5. Principal components were calculated using *sc.pp.pca* and *sc.pl.pca_variance_ratio* was used to determine the number of principal components used for clustering. The neighborhood graph was calculated using *sc.pp.neighbors* and then clustered using *sc.tl.leiden* with parameter resolution=0.4. Then the clusters were visualized using Uniform Manifold Approximation and Projection (UMAP) calculated by *sc.tl.umap*.

To annotate the major cell types, *sc.tl.rank_genes_groups* was applied to identify differentially expressed genes in each cluster using the Wilcoxon rank-sum test. The top significant differentially expressed genes were then manually reviewed. In addition, we checked each cluster using the known canonical markers such as EPCAM, KRT19, KRT14, ERBB2, ESR1 for epithelial cells, PECAM1, VWF for endothelial cells, DCN, COL1A1, COL1A2, COL3A1, CFD, and PRGFRB for CAFs, ACTA2, TAGLN, MCAM for perivascular cells, CD79A, CD79B for B cells, LYZ, IL1B, MSR1 for macrophages, JCHAIN, MZB1 for plasma cells, and CD3G, CD3D, IL7R, NKG7, GNLY, CD8A for T cells.

To investigate the heterogeneity of CAFs, cells from the subtype were extracted, and similar procedures were performed. In the clustering, we selected a high-resolution parameter (resolution_value=0.8) to obtain the fine-tuned subpopulations from this cell type. Differentially expressed genes were calculated for each CAF subpopulation against the remaining CAF subpopulations. The Wilcoxon rank-sum test was used for the statistical significance test as previously.

To infer the potential cell-cell communication network between clusters, CellChat (32) (version 1.6.1) was used to quantitatively measure networks following the tutorial. A CellChat object was created from the normalized expression matrix with the createCellChat function. After preprocessing data with identifyOverExpressedGenes, identifyOverExpressedInteractions, computeCommunProb, filterCommunication, and computeCommunProbPathway were used to calculate potential ligands–receptor interactions. Finally, the aggregated cell–cell communication network was calculated using the “aggregateNet” function.

The STRING database (https://string-db.org/) (33) was used to identify SFRP4-related protein-protein interactions. Gene set enrichment analyses (GSEA) were performed on the significantly differently expressed genes using GSEApy (https://github.com/zqfang/GSEApy) (34) with gene ontology (GO), Kyoto Encyclopedia of Genes and Genomes (KEGG) and MSigDB Hallmark signatures gene sets. Three GO terms biological processes (BPs), cellular components (CCs), and molecular functions (MFs) were included. We used a false discovery rate (FDR) of 0.25 as the significance criterion.

The copy number variation of single cells was inferred using InferCNV (https://github.com/broadinstitute/infercnv). A set of reference ‘normal’ cells were randomly selected from immune cells.

To infer the pseudotime of CAF subclusters, diffusion maps were calculated using sc.tl.diffmap. A putative initial cell was selected after studying the individual diffusion components and identifying the most extreme diffusion component in one dimension. The pseudotime was then calculated using sc.tl.dpt.

Bulk sequencing data was deconvoluted using Scaden (https://github.com/KevinMenden/scaden) (35). Scaden is a deep-learning based algorithm for cell type deconvolution of bulk RNA-seq samples. scRNA-seq data and bulk sequencing data were prepared as its instructions, and data simulations, data preprocessing, training, and prediction were run sequentially.

To visualize the spatial distribution of SFRP4+ CAF, we analyzed BC spatial transcriptomics data (V1_Breast_Cancer_Block_A_Section_1) downloaded from 10x Genomics. Cells with less than 5,000 counts or more than 35,000 counts were filtered. Cells with mitochondrial percentage >20 were also filtered. Genes detected in less than 10 cells were filtered. Then the counts were normalized, and log transformed. Highly variable genes were selected using *sc.pp.highly_variable_genes* with parameters flavor=“seurat”, n_top_genes=2000. Then *sc.pp.neighbors*, *sc.tl.umap*, and *sc.tl.leiden* were run to cluster the cells. *sc.pl.spatial* was used to visualize the expressions.

### 2.4 Association between gene expression and neoadjuvant chemotherapy response

Two datasets [GSE20194 (36), GSE20271 (37)] with neoadjuvant chemotherapy (NAC) response information were downloaded from the NCBI GEO database. GSE20194 had 278 samples and GSE20271 had 178 samples. The median expression of the target gene was used to split samples into high and low-expression groups in each cohort. The average gene expression in samples according to NAC response, pathological complete response (pCR) and residual disease (RD), was also compared.

### 2.5 Survival analysis

Python package lifelines (https://lifelines.readthedocs.io/en/latest/, version 0.26.4) (21) was used to perform Kaplan–Meier curve analysis, log-rank test in METABRIC dataset, and p < 0.05 was considered as significant. To validate the result, we further check the prognosis value of genes using an online database Kaplan-Meier Plotter (38).

### 2.6 Public dataset acquisition

Public single-cell gene expression datasets GSE164898 and GSE113197 were obtained from the GEO database (https://www.ncbi.nlm.nih.gov/geo/). Bulk sequencing data METABRIC was downloaded from cBioPortal. TCGA-BRCA including normal and tumor samples were acquired from the TCGA database (http://portal.gdc.cancer.gov/). The expression of BC cell lines was downloaded from the CCLE database.

### 2.7 Functional study of SFRP4+ CAF

#### 2.7.1 Cell culture and siRNA primers

The human BC cell line SK-BR-3 and HCC1937 were purchased from ATCC, and the CAF cell line was purchased from iCell Bioscience Inc with the product No. iCell-0091a. All cells were cultured in Dulbecco’s Modified Eagle Medium (Gibco) supplemented with 10% fetal bovine serum and 1% antibiotic (Gibco) at 37°C with 5% CO_2_. The cell lines were Mycoplasma-free and authenticated by PCR analysis monthly. Cells were used for no more than 12 months before being replaced. The SFRP4-targeting siRNA was transfected into the CAF cells using Lipofectamine 3000 reagent (Gibco). The siRNA sequences are listed in **Supplementary Table 1**.

#### 2.7.2 siSFRP4-CAFs conditioned media treatment

All cells were cultured in DMEM (Gibco) supplemented with 10% fetal bovine serum and 1% antibiotic (Gibco) at 37°C with 5% CO2. Cells were used for no longer than 12 months before being replaced. The cells can grow steadily after subcultured, when the siSFRP4 knockdown effect in BC cells is determined. The 6 mL of fresh media for replacement in 24h was collected and filtered, and then added to CAFs cells with 4 mL fresh media in 10 cm dish, and cultured for 48h. Repeat this procedure every other day.

#### 2.7.3 Western blot

The protein for western blotting was extracted using RIPA lysis buffer (Beyotime, China), then the protease inhibitor cocktail (Beyotime) and 0.1 mM PMSF (Beyotime) were added to the lysated protein. The protein concentrations were measured by a BCA assay kit (Beyotime), and thereafter, protein loading, electrophoresis, and membrane-transfer were operated according to the manufacturer’s instructions. Next, the primary antibody was used for incubating overnight and the second antibody was incubated for 2 hours. In the end, the targeted proteins were detected by the Beyo ECL Star Kit (Beyotime), and Bio-Rad Quantity One Software was used for quantification. Antibody’s information is listed in **Supplementary Table 1**.

#### 2.7.4 Quantification of mRNA by real-time PCR

Total RNA was isolated using Trizol reagent RNAiso Plus (Takara). The cDNA was reversely transcribed from the mRNA using the 5× Primescript RT Master Mix (Takara). Quantitative RT-PCR was performed using 2× SYBR Green Mix (Takara) in a Bio-Rad detection system. The content determination of targeted DNA was analyzed by quantitative real-time PCR. Primer sequences were listed in **Supplementary Table 1**.

#### 2.7.5 ELISA assay

The level of SFRP4 was estimated in different culture media by an ELISA kit (CUSABIO, China, Wuhan) according to the manufacturer’s instructions. The accuracy of the experimental results was based on three independent repeated experiments.

#### 2.7.6 Wound healing and Transwell invasion assays

For the wound healing assay, SK-BR-3 and HCC1937 cells were cultured in six-well plates. The wound in the center of the cell monolayer was scratched by a sterile plastic pipette tip, the wound was photographed at an indicated time of 0 and 24h. For the Transwell invasion assay, 5 × 104 suspended cells without FBS were plated on the upper chamber membranes (8 µm pore size, 6.5 mm diameter, Corning) coated with Matrigel (BD Biosciences), and incubated in 500 µl medium with 10% FBS. The invasive ability was evaluated by the stained invasive cells with crystal violet. Stained cells were photographed and quantified in five randomly selected areas and six independent experiments were performed.

## 3 Results

### 3.1 scRNA-seq identified eight major cell types in breast cancer

**Figure 1:**
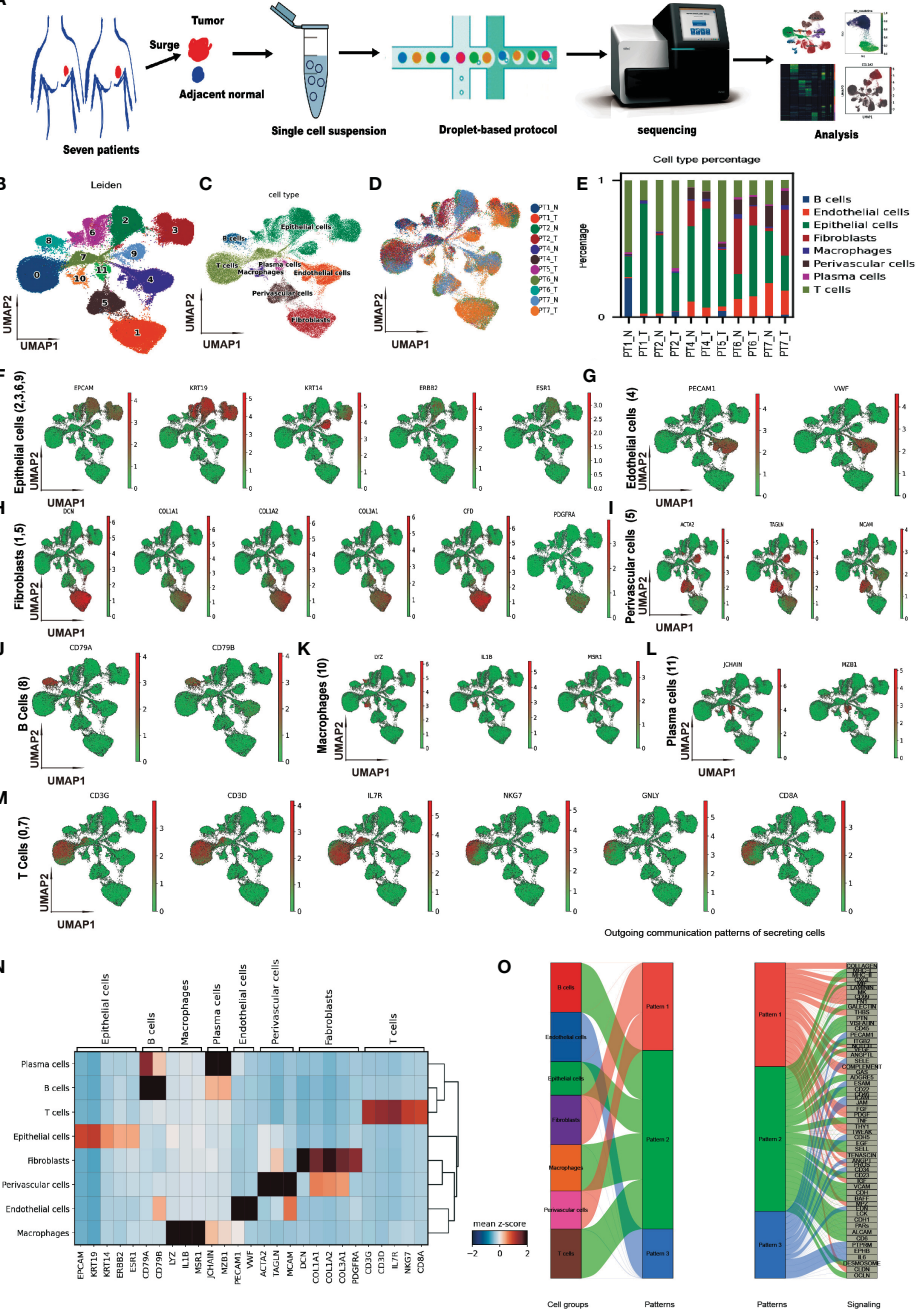
Single-cell transcriptome profiling of breast cancer and adjacent non-tumor tissues. **(A)** Schematic diagram of the study design and workflow. **(B)** Uniform Manifold Approximation and Projection (UMAP) plot of 71,773 cells from eleven samples, colored by 12 Leiden clusters. **(C)** UMAP plot of cells, colored by eight annotated cell types. **(D)** UMAP plot of cells, colored by samples. **(E)** Bar plot of cell type proportions across samples. **(F–M)** UMAP plots of selected marker genes for epithelial, endothelial, CAF, perivascular, B, macrophage, plasma, and T cells. **(N)** Matrix plot of average expression of marker genes in cell types **(O)** River plot of out-going communication patterns in eight cell types.

To characterize cell expression heterogeneity in BC, we performed droplet-based single-cell transcriptome profiling (scRNA-seq, 10× Genomics Chromium system) on fresh tumors and adjacent non-tumor tissues from seven breast cancer (BC) patients after surgery (**Figure 1A**). The clinical and pathological information of the patients was summarized in **Table 1**. An average of 5,752 cells per sample was sequenced with 221,292 average reads per cell. After quality control (Methods), eleven of fourteen samples and 71,773 cells were retained, of which 60.4% were from tumor samples.

**Table 1:** Sample clinical information.

Individual ID	Age	Pathologic stage	ER/PR/HER2 (IHC)	Ki67
PT1	47	T1N1 (IIA)	+/+/-	10%+
PT2	47	T2N0 (IIIA)	+/-/+	30%+
PT3	69	T2N1 (IIA)	+/+/+	25%+
PT4	54	T2N0 (IA)	+/-/+	10%+
PT5	40	T2N0 (IIIA)	+/+/+	20%+
PT6	44	T2N1 (IIA)	+/+/-	30%+
PT7	62	T1N1 (IIA)	+/+/-	15%+

The count matrix was normalized, the dimensionality reduction was performed by the principal component analysis, and the graph-based Leiden clustering was applied to classify cells into twelve transcriptionally distinct clusters and the clusters were visualized using UMAP (**Figure 1B**). We annotated the clusters into eight major cell types based on the expression of canonical cell type marker genes and the top differentially expressed genes of each cluster (**Figure 1C**). The sample level distribution of cells was shown in **Figure 1D**.

We observed heterogeneity in the cell type compositions across samples, even from the same individual (**Figure 1E**). The cell type markers included EPCAM, KRT19, KRT14, ERBB2, ESR1 for epithelial cells (**Figure 1F**), PECAM1, VWF for endothelial cells (**Figure 1G**), DCN, COL1A1, COL1A2, COL3A1, CFD, and PRGFRA for cancer-associated fibroblasts (CAFs) (**Figure 1H**), ACTA2, TAGLN, MCAM for perivascular cells (**Figure 1I**), CD79A, CD79B for B cells (**Figure 1J**), LYZ, IL1B, MSR1 for macrophages (**Figure 1K**), JCHAIN, MZB1 for plasma cells (**Figure 1L**), and CD3G, CD3D, IL7R, NKG7, GNLY, CD8A for T cells (**Figure 1M**). A matrix plot of expressions of these markers across the cell types is shown in **Figure 1N**. In addition to the CAF markers mentioned above, GSN, LUM, APOD, and FBLN1 were also highly expressed.

To investigate the genetic alteration in CAF, we analyzed the copy number variation (CNV) using InferCNV. No significant CNV was detected in CAFs (**Supplementary Figure 1**), which was consistent with the previous report (39). Furthermore, we analyzed the cell-cell communications between major cell types using CellChat. We found complex interactions between CAF and other cell types (**Supplementary Figure 2**). Three outgoing patterns were identified in the communications of secreting cells, and CAF was dominant in pattern 1, which included interactions of COLLAGEN, CXCL, LAMININ, et al. (**Figure 1O**). This is consistent with the functional of fibroblasts in cell matrix.

### 3.2 Heterogeneity of cancer-associated fibroblasts in breast cancer

**Figure 2:**
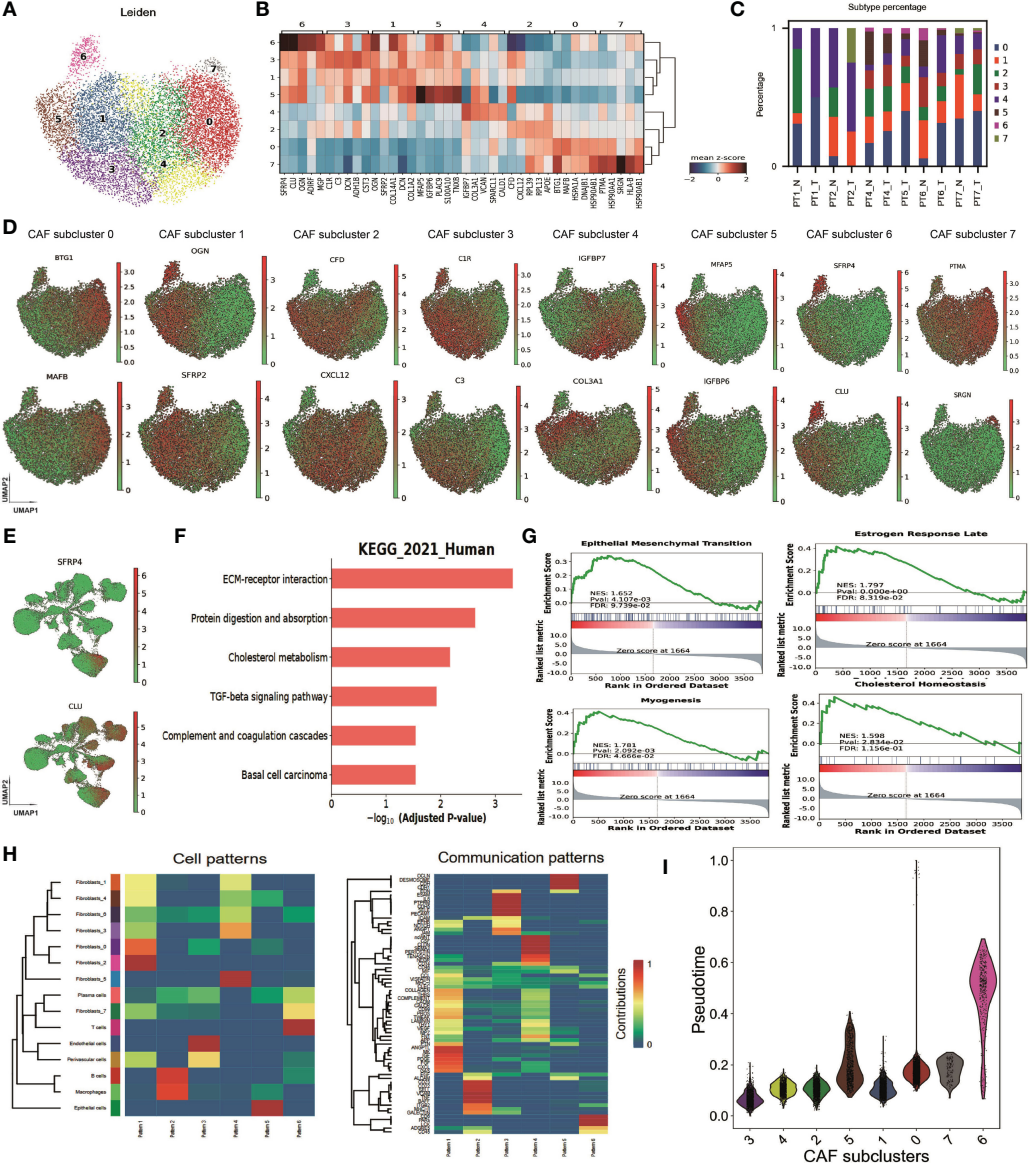
Heterogeneity of cancer-associated fibroblasts in breast cancer. **(A)** Uniform Manifold Approximation and Projection (UMAP) plot of cells showing subtypes in CAFs. **(B)** Matrix plot of marker genes in CAF subtypes averaged from the scRNA-seq data. **(C)** Bar plot of CAF subtype percentage across samples. **(D)** UMAP plot of cells, colored by maker gene expression in each CAF subtype. **(E)** UMAP plot of all cells, colored by the expression of gene SFRP4, and CLU. **(F)** Kyoto Encyclopedia of Genes and Genomes (KEGG) enrichment of CAF marker genes. **(G)** Gene Set Enrichment Analysis (GSEA) enrichment on MSigDB Hallmark signatures. **(H)** Heatmap showing the cell and communication patterns. **(I)** Distribution of pseudotime values in each CAF subtype. SFRP4+ CAF, corresponding to CAF subcluster 6.

To explore the cellular heterogeneity within CAFs, we normalized, reduced the dimensionality, and clustered 11,831 CAF cells using a finely tuned pipeline, described in Methods. Eight CAF subtypes have been identified (**Figure 2A**). The expressions of the top five marker genes for each subtype are shown in **Figure 2B**. CAF subtypes 1, 3, 5, and 6 clustered together, while the remaining subclusters 0, 2, 4, and 7 formed another group. The percentages of each CAF subcluster across samples were shown in **Figure 2C**. We named each CAF subtype according to their most distinctive marker gene: BTG1+ CAF, corresponding to subtype 0; OGN+ CAF, corresponding to subtype 1; CFD+ CAF, corresponding to subtype 2; C1R+ CAF, corresponding to subtype 3; IGFBP7+ CAF, corresponding to subtype 4; MFAP5+ CAF, corresponding to subtype 5; SFRP4+ CAF, corresponding to subtype 6; and PTMA+ CAF, corresponding to subtype 7. The expression of the top 2 marker genes for each subtype were shown in **Figure 2D**. We found that SFRP4+ CAF was the most separated from the other subtypes in the UMAP plot (**Figure 2A**). SFRP4 was specifically expressed in this subtype, while CLU was also expressed in other cell types (**Figure 2E**).

We performed differential expression gene analyses between CAFs and other cell types to investigate the potential function of CAFs. The differentially expressed genes involved extracellular matrix organization, collagen fibril organization, transforming growth factor beta binding, and ECM-receptor interaction, according to GO and KEGG enrichment (**Figure 2F** and **Supplementary Figure 3**). GSEA enrichment on MSigDB Hallmark signatures showed that they associated with estrogen response late, myogenesis, epithelial-mesenchymal transition, and cholesterol homeostasis pathways (**Figure 2G**). These findings suggest that CAFs may play a key role in modulating the tumor microenvironment and promoting BC progression and metastasis.

We then analyzed the cell-cell communication patterns between CAF subtypes and other cell types. We found that CAF subtypes were enriched in pattern 1 and pattern 4 while T cells enriched in pattern 6, B cell/macrophage enriched in pattern 2, endothelial cells in pattern 3 and epithelial cells in pattern 5 (**Figure 2H**). Pattern 1 involved signaling pathways of COLLAGEN, CD99, MK, CXCL et al. while pattern 4 involved CLDN, CD34, ncWNT, et al. These patterns imply that CAFs may influence the immune response and the epithelial-mesenchymal transition of tumor cells. BTG1+ CAF, OGN+ CAF, CFD+ CAF, IGFBP7+ CAF were in pattern 1, and C1R+ CAF, MFAP5+ CAF were in pattern 4 (**Supplementary Figure 4**). Interestingly, SFRP4+ CAF had a weak signal in four patterns, suggesting that it may have a different communication mode from the other subtypes. A diffusion pseudotime analysis suggested that SFRP4+ CAF was in a distinct path from the other subtypes (**Figure 2I**). The distinct cell-cell communication patterns and different pseudo development trajectory indicate that SFRP4+ CAF may have a unique biological function and role in breast cancer.

### 3.3 SFRP4 CAF+ validation and potential functionality

**Figure 3:**
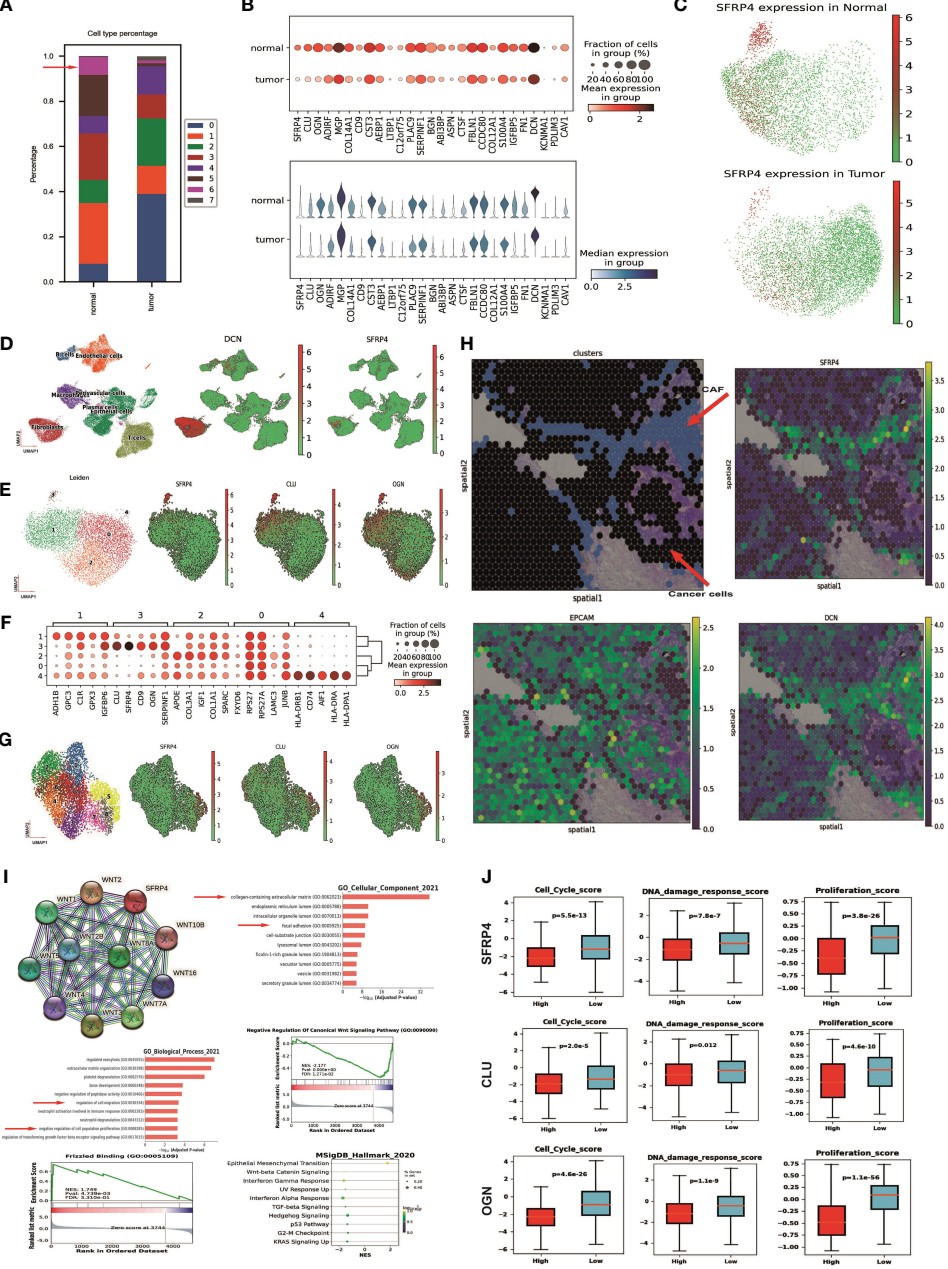
SFRP4 CAF+ validation and potential functionality. **(A)** Percentages of each CAF subtype across samples. **(B)** Dot plot and violin plot of SFRP4+ CAF marker genes. Most of them are highly expressed in normal samples. **(C)** UMAP plot of SFRP4 expression in normal and tumor samples. **(D)** UMAP plot of cell types and expressions of the gene DCN and SFRP4 using dataset GSE164898. **(E)** UMAP plot of CAF subtypes and the expression of gene SFRP4, CLU, OGN using dataset GSE164898. **(F)** Dot plot of expression of the top five marker genes in each CAF subtypes using dataset GSE164898. **(G)** UMAP plot of CAF subtypes and the expression of gene SFRP4, CLU, OGN using dataset GSE225600. **(H)** Regional plot of clusters and expressions of SFRP4, EPCAM, and DCN in a spatial transcriptome dataset. **(I)** Protein-protein interaction of SFRP4 from STRING-db; GO and GSEA pathway enrichment of SFRP4+ CAF marker genes. **(J)** Box plot of cell cycle scores, DNA damage response scores, and cell proliferation scores according to the expression of marker genes SFRP4, CLU, OGN in TCGA-BRCA cohort.

We compared the percentages of each CAF subtype between the normal and tumor samples using scRNA-seq data. We found that CAF subtypes 0 (BTG1+ CAF), 2 (CFD+ CAF), and 4 (IGFBP7+ CAF) were more abundant in the tumor than the normal, while CAF subcluster 1 (OGN+ CAF), 3 (C1R+ CAF), 5 (MFAP5+ CAF), and 6 (SFRP4+ CAF) were more abundant in the normal than the tumor (**Figure 3A**). Most of the marker genes of SFRP4+ CAF (subcluster 6) were highly expressed in the normal samples (**Figure 3B**). Of the top 30 SFRP4+ CAF marker genes, eleven genes (11/30 = 36.7%) were significantly upregulated in the normal samples (**Supplementary Figure 5**, Student’s T test, p<0.001). The expression of SFRP4 in normal samples and tumor samples were shown in **Figure 3C**.

We then confirmed the expression specificity of SFRP4 in SFRP4+ CAF by reanalyzing several publicly deposited datasets. GSE75688 (40), a BC single-cell dataset generated by full-length single-cell RNA sequencing, also demonstrated that SFRP4 was only expressed in some CAF cells. GSE113197 (41), another BC scRNA-seq dataset generated from cells first sorted using epithelial cell surface markers, confirmed that SFRP4 was not expressed in epithelial cells. GSE164898 (42), another scRNA-seq dataset in the breast, confirmed the existence of SFRP4+ CAF. The eight cell types were clustered and annotated using the same pipeline for the in-house dataset (**Figure 3D** left). Marker DCN was highly expressed in CAF cluster (**Figure 3D** middle) and SFRP4 was expressed in some CAFs (**Figure 3D** right). Further CAFs were fine clustered into five subpopulations and genes SFRP4, CLU, OGN were highly expressed in CAF subpopulation 3 (**Figure 3E**). The marker gene expressions of CAF subpopulations were shown in **Figure 3F**. We further confirm the existence of SFRP4+ CAF using a BC scRNA-seq data GSE225600. SFRP4 and CLU were highly expressed in the CAF subcluster 5 (**Figure 3G**). Then we accessed the expression distribution of SFRP4 in BC tissue using a spatial transcriptome dataset from Illumina. We found that the distribution of SFRP4 was aligned with DCN, a CAF marker gene, and separated from EPCAM, an epithelial cell marker gene (**Figure 3H**; **Supplementary Figure 6**). In addition, we compared the expression of SFRP4+ CAF marker genes in normal and tumor samples using data from the TCGA-BRCA cohort and found that most of the marker genes of SFRP4+ CAF were significantly down-regulated in the tumor samples (**Supplementary Figure 7**), which was consistent with our scRNA-seq results.

We investigated the possible function of SFRP4+ CAF by constructing a protein interaction network from STRING-db. We found that SFRP4 could directly interact with several WNT components (**Figure 3I**). GO enrichment of the top marker genes of SFRP4+ CAF showed that they were involved in collagen-containing extracellular matrix and focal adhesion in cellular component items, and regulation of cell migration and negative regulation of cell population proliferation in biological process items (**Figure 3I**). GSEA enrichment of genes found the enrichment of the WNT signaling pathway and frizzled binding. The MSigDB hall markers also found enrichment of the Wnt-beta catenin signaling pathway (**Figure 3I**). In the bulk sequencing data, we stratified the tumor samples into two groups according to the median expression of the marker genes (SFRP4, CLU, OGN). We found that samples with low expression of these genes have significantly higher cell cycle scores, DNA damage response scores, and cell proliferation scores (**Figure 3J**). These findings suggest that SFRP4+ CAF may play a key role in modulating the tumor microenvironment and inhibiting tumor progression.

We explored the interaction of SFRP4+ CAF with WNT signaling by checking the expression level of the WNT genes in our scRNA-seq data and CCLE database. We found that WNT5A was highly expressed in our scRNA-seq dataset using all epithelial cells (**Supplementary Figure 8**) and BC cell lines (**Supplementary Figure 9**). Therefore, we hypothesized that SFRP4+ CAF may inhibit BC progression through WNT5A in the WNT pathway.

### 3.4 WNT-β-catenin activation induced by SFRP4 silencing in CAFs promote migration

**Figure 4:**
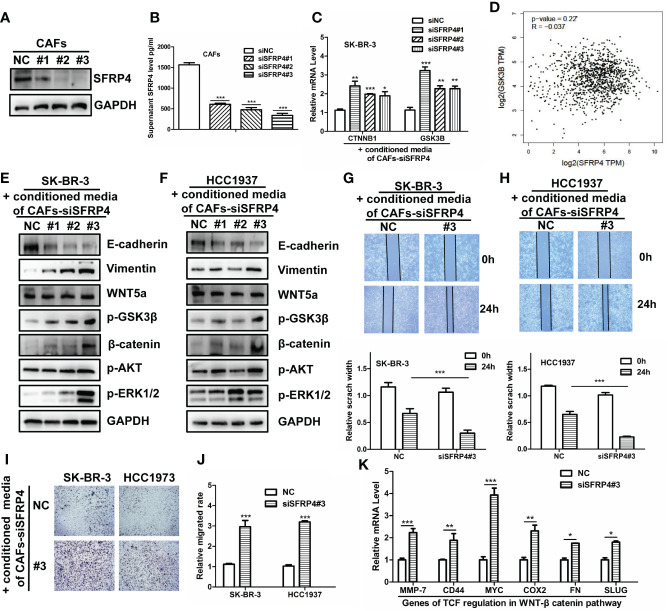
SFRP4 silencing in CAFs activates WNT signaling and promotes BC cell migration. **(A)** The expressions of SFRP4 were successfully silenced by three sequences of siRNA. **(B)** SFRP4 protein level in the conditioned media was quantified by ELISA and showed a significant reduction after siRNA silencing. **(C)** SK-BR-3 cells were treated with the conditioned media of CAFs-siSFRP4 and showed increased mRNA expression of CTNNB1 and GSK3B, two genes involved in canonical WNT signaling. **(D)** SFRP4 and GSK3B mRNA levels were weak negative correlated in the GEPIA database. **(E, F)** The detection of protein level of E-cadherin, Vimentin, WNT5a, p-GSK3β, β-catenin, p-AKT and p-ERK1/2 in SK-BR-3 and H1937 cancer cells when treatment with conditioned media of CAFs-siSFRP4. **(G, H)** The wound healing assay showed that the conditioned media of CAFs-siSFRP4 enhanced the capability of invasiveness of SK-BR-3 and H1937 cells, as evidenced by the reduced scratch width. **(I, J)** The migration assay showed that the conditioned media of CAFs-siSFRP4#3 significantly increased the number of SK-BR-3 and H1937 cells that passed through the membrane, indicating increased the capability of migration. **(K)** The mRNA expression of TCF downstream proteins, MMP-7, CD44, MYC, COX2, FN, and SLUG was up-regulated by SFRP4 silencing in CAFs. "*" represents p < 0.05, "**" represents p < 0.01, and "***" represents p < 0.001.

We hypothesized that SFRP4+ CAFs secrete SFRP4 and inhibit the WNT signal pathway in BC cells. SFRP4 is a secreted frizzled-related protein that interferes with WNT-Fz interactions and thus inhibits the WNT pathway (43, 44). Our analysis suggested that SFRP4+ CAFs may contribute to cell proliferation and migration. To assess our hypothesis, we silenced SFRP4 in cancer-associated fibroblasts (CAFs) using three siRNAs and examined the effects on BC cells. We confirmed the reduced expression of SFRP4 in CAFs (**Figure 4A**). ELISA experiment demonstrated that the level of conditioned media SFRP4 protein in cell-culture dish decreased when siSFRP4 silencing (**Figure 4B**). We then treated SK-BR-3 cells with the conditioned media of CAFs-siSFRP4 and observed an up-regulation of CTNNB1 and GSK3B mRNA, which are involved in WNT signaling (**Figure 4C**). This was consistent with a weak negative correlation between SFRP4 and GSK3B mRNA levels using the GEPIA database (**Figure 4D**). The protein from SK-BR-3 and H1937, treated with conditioned media of CAFs-siSFRP4, were used for detecting the activation of WNT signaling. In addition, SFRP4 silencing in CAFs induced EMT phenotypic transition, as evidenced by increased vimentin and decreased E-cadherin expression. However, we did not observe any changes in WNT5a. The level of GSK3β phosphorylation and β-catenin accumulation were significantly up-regulated in SK-BR-3 and H1937 BC cells after treatment with CAFs-siSFRP4 conditioned media. Moreover, we detected an activation of AKT and ERK1/2 pathways (**Figures 4E, F**). The SK-BR-3 and HCC1937 cells had a higher cell proliferation rate when co-culture with siSFRP4-CAFs compared with the control group (**Supplementary Figure 10**). These results indicate that SFRP4 silencing in CAFs activates WNT signaling pathway. Furthermore, the healing and migration assay demonstrated that the capability of invasiveness and migration of BC cells were significantly increased when treatment with the conditioned media of CAFs-siSFRP4 (**Figures 4G–J**). It is known that WNT-β-catenin activation enhanced the transcriptional function of TCF, so we also measured the mRNA expression of several downstream targets of TCF, such as MMP-7, CD44, MYC, COX2, FN, and SLUG, and found that they were generally increased after SFRP4 silencing in CAFs (**Figure 4K**). In conclusion, our study showed that SFRP4+ CAFs inhibit the WNT-β-catenin pathway in BC cells by secreting SFRP4. Silencing SFRP4 in CAFs induced EMT and increased the metastatic potential of BC cells.

### 3.5 Potential clinical impact of SFRP4+ CAF

**Figure 5:**
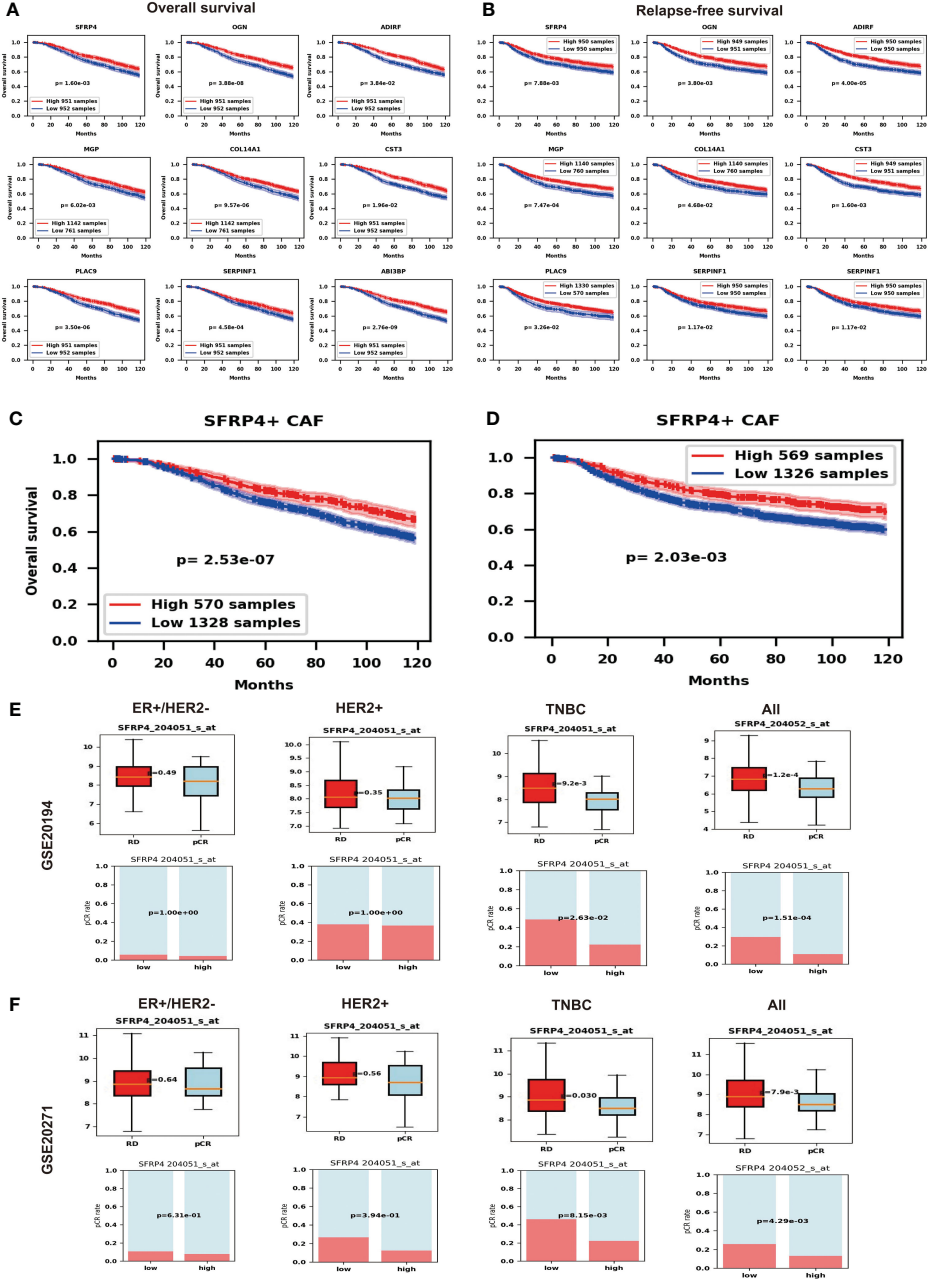
Potential impact of SFRP4+ CAF. **(A)** Nine marker genes of SFRP4+ CAF consistently predict better overall survival (OS). **(B)** Nine marker genes of SFRP4+ CAF consistently predict better relapse-free survival (RFS). **(C)** We show that SFRP4+ CAF composition can also predict the OS. **(D)** We show that SFRP4+ CAF composition can also predict the RFS. **(E)** The pCR group has significantly lower SFRP4 expression than the RD group and the low SFRP4 group has a significantly higher pCR rate than the high SFRP4 group in TNBC and all BC using dataset GSE20194. **(F)** The pCR group has significantly lower SFRP4 expression than the RD group and the low SFRP4 group has a significantly higher pCR rate than the high SFRP4 group in TNBC and all BC using dataset GSE20271.

Since SFRP4+ CAF can modulate the WNT pathway, it may influence the prognosis of BC. We show the associations by performing Kaplan–Meier curve survival analysis and log-rank test in the METABRIC dataset. From the top fifteen marker genes, nine of them - SFRP4, OGN, ADIRF, MGP, COL14A1, CST3, PLAC9, SERPINF1 and ABI3BP - consistently predict better overall survival (OS) (**Figure 5A**) and relapse-free survival (RFS) (**Figure 5B**) while higher expressed. We then validate the prognosis of these genes in an online database Kaplan-Meier Plotter and find that except for ADIRF did not exist, and PLAC9 is not significant in the Kaplan-Meier Plotter, others are consistently significant in the Kaplan-Meier Plotter (**Supplementary Figure 11**). Further, we deconvolute the METABRIC dataset using Scaden. We show that SFRP4+ CAF composition can also predict the OS (**Figure 5C**) and RFS (**Figure 5D**), the higher the percentage of SFRP4+ CAF, the better the OS and RFS.

The modified tumor microenvironment may also contribute to drug therapy. Therefore, we further check the gene expression of SFRP4 and the BC NAC outcomes. To our surprise, the pCR group has significantly lower SFRP4 expression than the RD group while the low SFRP4 group has a significantly higher pCR rate than the high SFRP4 group in TNBC subtypes and in all BC samples (**Figure 5E**). We further validate the result using an independent dataset (**Figure 5F**). This result suggests that SFRP+ CAF may have an impact on the NAC outcomes.

## 4 Discussion

In this study, we used scRNA-seq to explore the heterogeneity of CAFs in BC. We analyzed paired tumor and adjacent normal samples from seven BC patients and identified eight CAF subtypes with distinct gene expression profiles. One subtype, SFRP4+ CAFs, was novel and more abundant in normal samples. We found that SFRP4+ CAFs can inhibit BC cell migration and EMT through the canonical WNT pathway. Our findings reveal the diversity and dynamics of CAFs in BC and suggest that SFRP4+ CAFs may predict prognosis and chemotherapy response.

We classified the cells into eight major cell types based on their marker gene expression. The CAF cluster had high expressions of DCN, COL1A1, COL1A2, COL3A1, CFD, and PDGFRA, and low expression of other cell type markers. These markers are consistent with previous publications (26, 45, 46). Fine-tune clustering in CAF identified eight subtypes: BTG1+ CAF, OGN+ CAF, CFD+ CAF, C1R+ CAF, IGFBP7+ CAF, MFAP5+ CAF, SFRP4+ CAF, and PTMA+ CAF. These subtypes showed distinct gene expression profiles and functions. BTG1 was reported to protect cells from oncogenic transformation (47); IGFBP7+ CAF was reported to promote gastric cancer (48); CAF-derived MFAP5 activated cell growth and migration in oral tongue squamous cell carcinoma via activation of MAPK and AKT pathways (49). The marker gene of myCAF ACTA2, was significantly higher expressed in IGFBP7+ CAF (p_adj_=1.75e-28); this suggest IGFBP7+ CAF may be long to myCAFs. The marker gene of iCAFs IL6 was significantly higher expressed in OGN+ CAF (p_adj_=5.90e-20), suggesting OGN+ CAF may be long to iCAF. The marker gene of apCAF CD74 was significantly higher expressed in PTMA+ CAF (p_adj_=2.22e-13), suggesting it may belong to apCAF. Based on the expression of marker genes, SFRP4+ CAF did not belong to myCAF, iCAF or apCAF. Meanwhile, in SFRP4+ CAFs, S100A4, CAV1, and FAP were upregulated while ACTA2 and PDGFRB were downregulated suggested that SFRP4+ CAF did not belong to any of four CAF subsets proposed by Costa (12, 20–22). This also suggests that classification based on six markers may not capture the heterogeneity of CAFs. Functional enrichment analysis showed that the CAF markers were related to extracellular matrix organization, collagen-containing extracellular matrix, transforming growth factor beta binding, and ECM-receptor interaction; this is consistent with CAF known functions (3, 18, 50).

We compared normal and tumor samples and found that SFRP4+ CAFs were more abundant in normal samples. SFRP4+ CAFs were also distinct from other CAF subtypes in clustering analysis, communication patterns, and pseudotime. We validated their existence in several public datasets. The GO enrichment of their marker genes showed that they participated in regulating cell migration and proliferation. Co-culture of siSFRP4+ CAFs and BC cell lines, we showed that SFRP4+ CAFs inhibited the WNT-β-catenin pathway in BC cells by secreting SFRP4. Silencing SFRP4 in CAFs induced EMT and increased BC cell migration through WNT signaling mechanism. The WNT signaling pathway regulates various cellular processes, such as cell proliferation, differentiation, and motility (51). Previous studies reported that SFRP4 modulated EMT, cell migration, and WNT signaling in ovarian cancer cells (52) and promoted apoptosis in glioblastoma cells (53). Other marker genes of SFRP4+ CAFs, such as OGN and CLU, also influenced cell proliferation and invasion (54) and cancer initiation (55), respectively. These findings suggested that SFRP4+ CAFs may protect against BC progression and that targeting SFRP4 may have therapeutic implications. However, further studies are needed to validate these results and to explore the molecular mechanisms of SFRP4+ CAFs and BC cells interaction.

We investigated the clinical impact of SFRP4+ CAFs by correlating their marker gene expression with BC patient prognosis. We found that high expression of SFRP4+ CAF markers was associated with better OS and RFS. The composition of SFRP4+ CAFs also predicted better prognosis after deconvolution of the METABRIC dataset. Moreover, we found that SFRP4 had value in predicting NAC response. As we discussed above, SFRP4 and other marker genes of SFRP4+ CAFs could inhibit cell proliferation and migration, which may explain why the high expression of these markers was linked to better prognosis. However, we also found that high SFRP4 expression was associated with a lower pCR rate than low SFRP4 expression. This contradicted the report that SFRP4 conferred chemo-sensitization and improved chemotherapeutic efficacy (56, 57). This may be complicated by other genes in SFRP4+ CAFs, such as CLU, which was reported to confer resistance to chemotherapy in BC (58). SFRP4+ CAFs may also affect immunotherapy, since FAP was reported as a target for immunotherapy (59, 60). These suggested the potential clinical impact of SFRP4+ CAFs.

Our study had some limitations. First, we could not separate SFRP4 from fresh tissue because it was a secreted protein. Second, we focused on the function of SFRP4 in SFRP4+ CAFs because it was the most significantly changed and specific gene. However, other marker genes may also contribute to the biological functions. Third, we analyzed only seven BC patients with different clinical and pathological characteristics. Therefore, our results may not represent the general BC population.

## 5 Conclusion

Using scRNA-seq, we discovered a new CAF subtype, SFRP4+ CAFs, that was more common in normal than tumor samples and inhibited BC cell migration by secreting SFRP4. SFRP4+ CAFs also indicated better survival and chemotherapy response in BC patients. Our study showed the complexity of CAFs in BC and their potential role in preventing BC progression and improving BC treatment.

## Code availability

The analysis codes were uploaded to GitHub https://github.com/luwening/singlecellanalysis.

## Data availability statement

Publicly available datasets were analyzed in this study. This data can be found here: https://www.ncbi.nlm.nih.gov/geo/, GSE20194, GSE20271, GSE164898, and GSE113197. The METABRIC dataset was downloaded from cBioPortal (https://www.cbioportal.org/), the TCGA-BRCA dataset was downloaded from the GDC database (http://portal.gdc.cancer.gov/), expressions of BC cell lines were downloaded from CCLE database (https://sites.broadinstitute.org/ccle/). The single-cell expression data generated in this study was deposited in The National Genomics Data Center (NGDC, https://ngdc.cncb.ac.cn/) with accession ID OMIX006159.

## Ethics statement

The studies involving humans were approved by Institutional Review Board of The Second Hospital of Shenzhen, China. The studies were conducted in accordance with the local legislation and institutional requirements. The participants provided their written informed consent to participate in this study.

## Author contributions

LN: Writing – review & editing, Writing – original draft, Methodology, Funding acquisition, Formal analysis, Conceptualization. CQ: Writing – review & editing, Methodology, Formal analysis, Conceptualization. YW: Writing – review & editing, Methodology, Formal analysis, Conceptualization. ZW: Writing – review & editing, Methodology, Formal Analysis, Conceptualization. PY: Writing – review & editing, Methodology, Formal analysis, Conceptualization. NX: Writing – review & editing, Supervision, Project administration, Funding acquisition, Conceptualization.

## Supplementary material

Supplementary Table 1 The siRNA sequences, antibody information, and primers sequences.
