# Supplementary material for: scRNA-seq characterizing the heterogeneity of fibroblasts in breast cancer reveals a novel subtype SFRP4+ CAF that inhibits migration and predicts prognosis
Source: Front Oncol. 2024 Apr 15;14:1348299. doi: 10.3389/fonc.2024.1348299 (PMC11056562; doi:10.3389/fonc.2024.1348299)
Supplement: Supplementary file 1 [file Image_1.pdf]

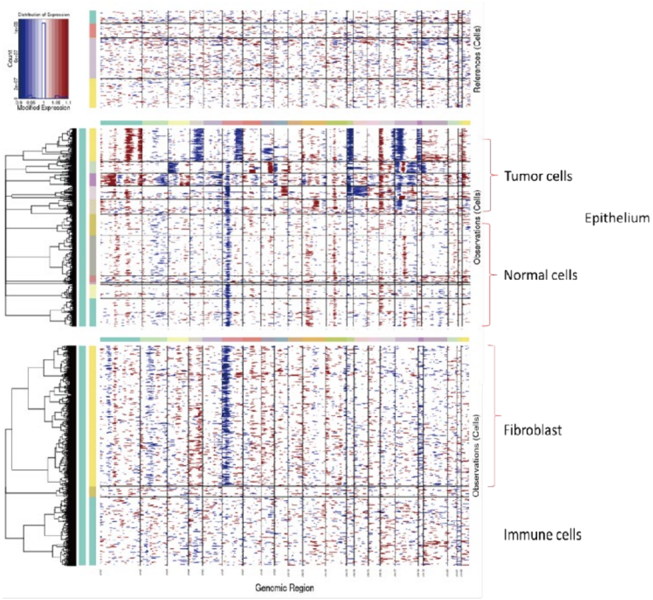

Supplemental Figure 1 Heatmap of inferred CNV of cells in different cell types.

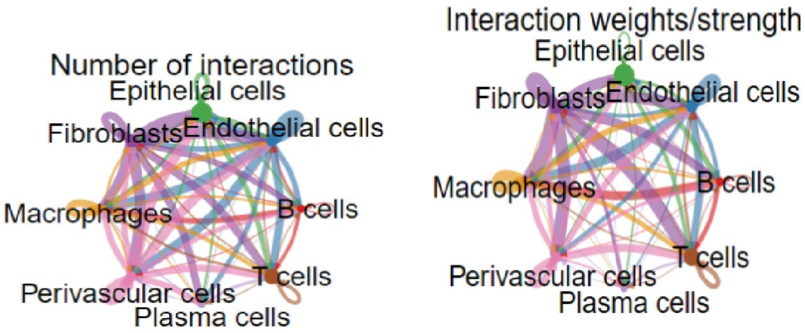

Supplemental Figure 2 Network diagram of inferred cell-cell interactions using CellChat.

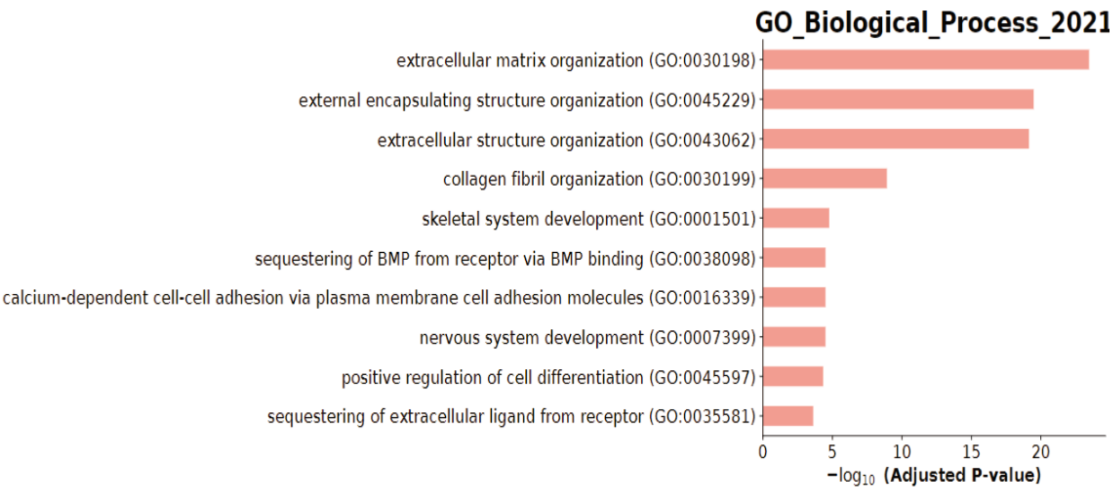

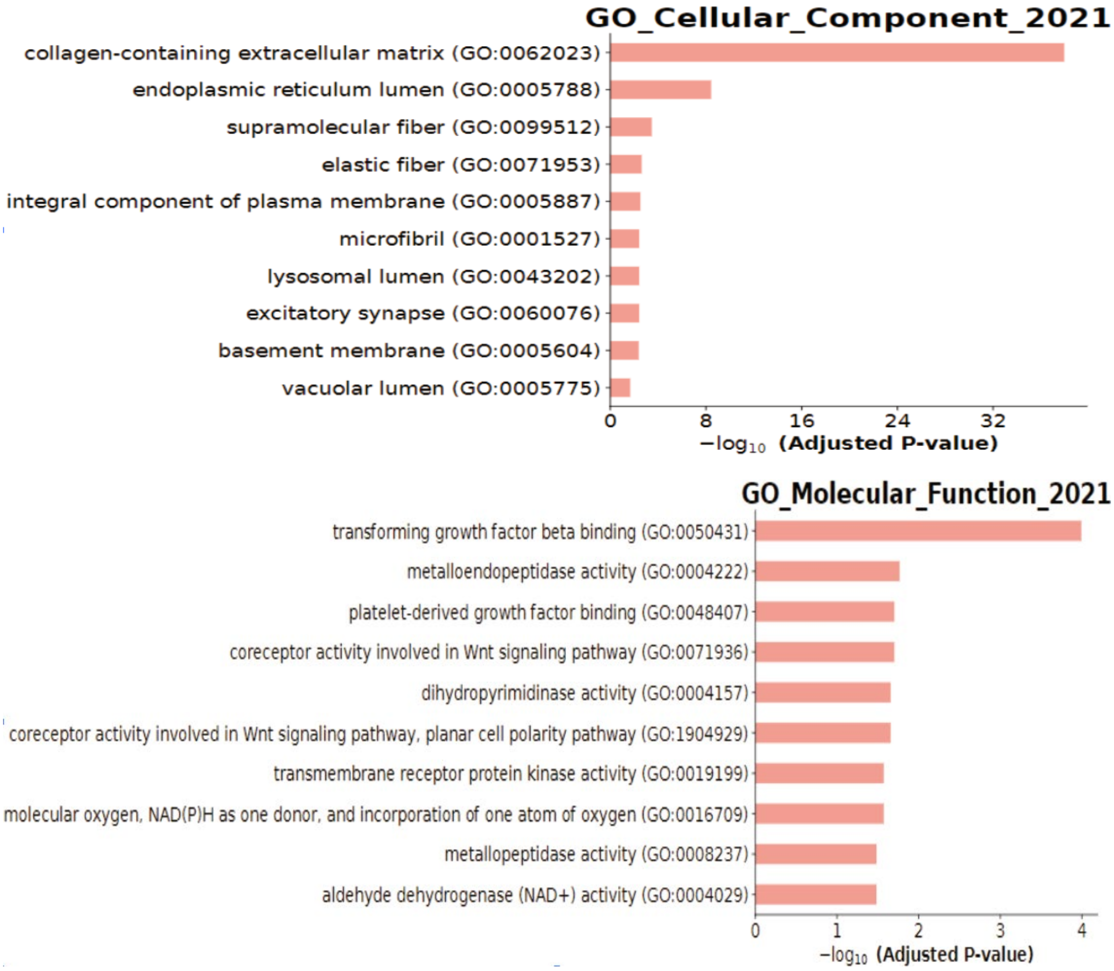

Supplemental Figure 3 Gene Ontology (GO) enrichment of CAF marker genes.

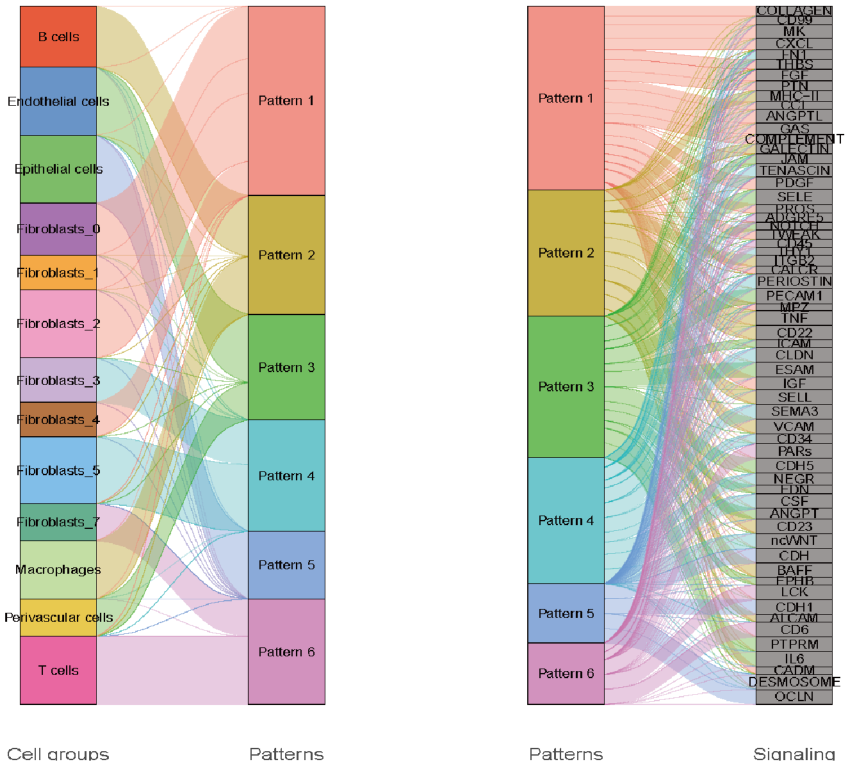

Supplemental Figure 4 River plot of out-going pattern in CAF subtypes and other cell types.

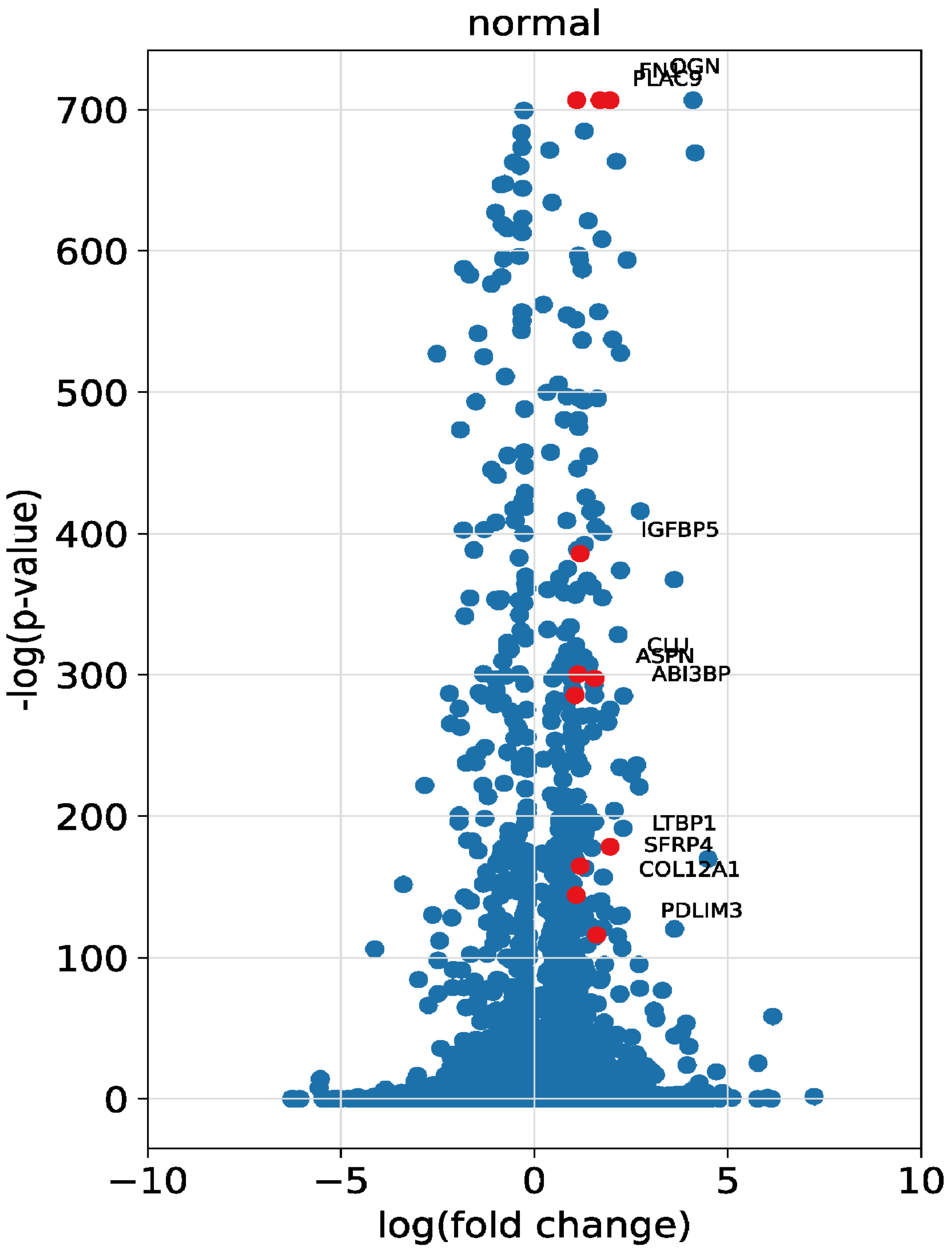

Supplemental Figure 5 Volcano plot showing that eleven marker genes of SFRP4+ CAF are significantly highly expressed in adjacent normal samples.

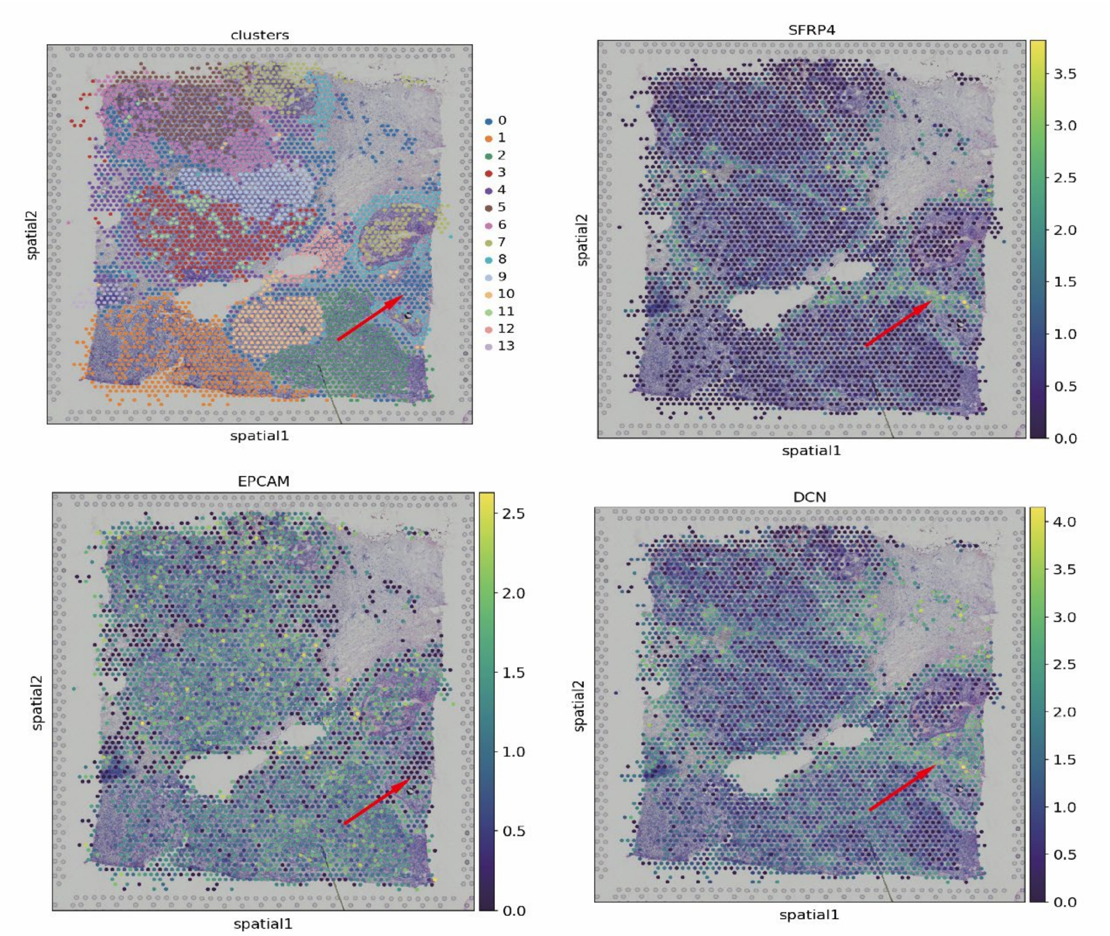

Supplemental Figure 6 Plot of clusters and expressions of SFRP4, EPCAM, and DCN in a spatial transcriptome dataset.

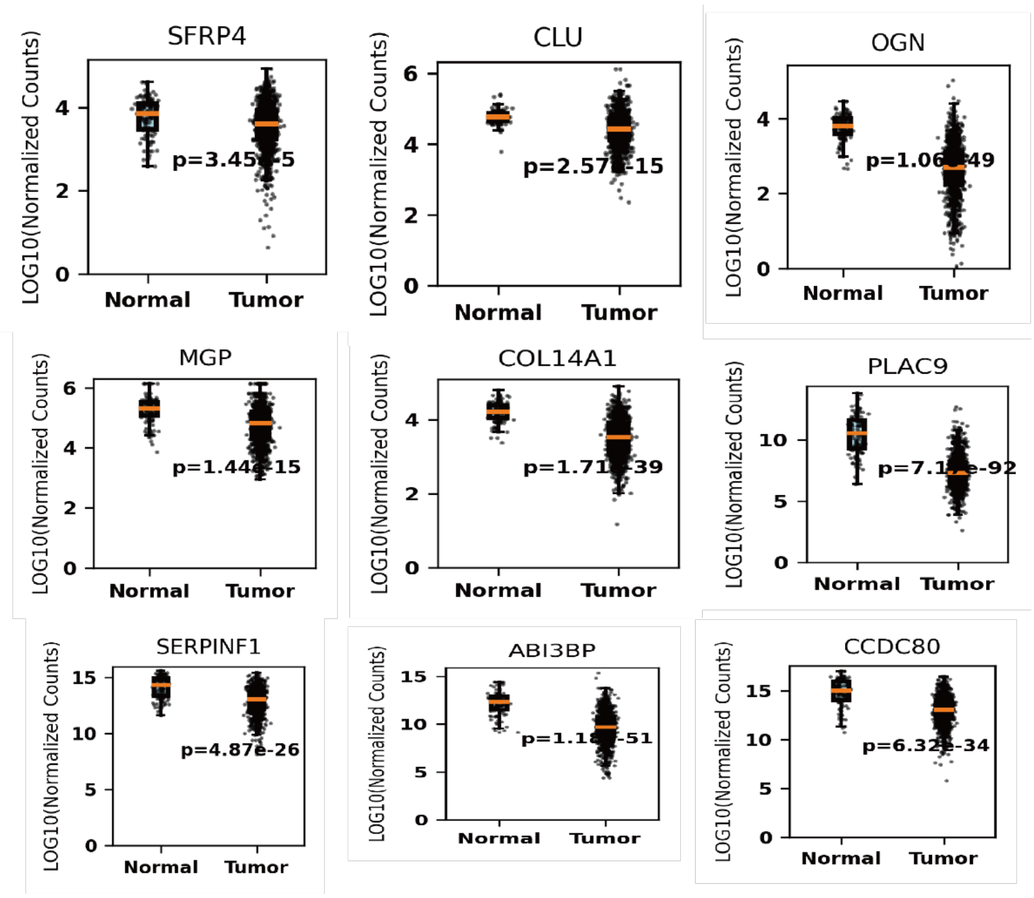

Supplemental Figure 7 Box plot of expression of nine marker genes (SFRP4, CLU, OGN, MGP, COL14A1, PLAC9, SERPINF1, ABI3BP, CCDC80) in normal and tumor in the TCGA-BRCA cohort.

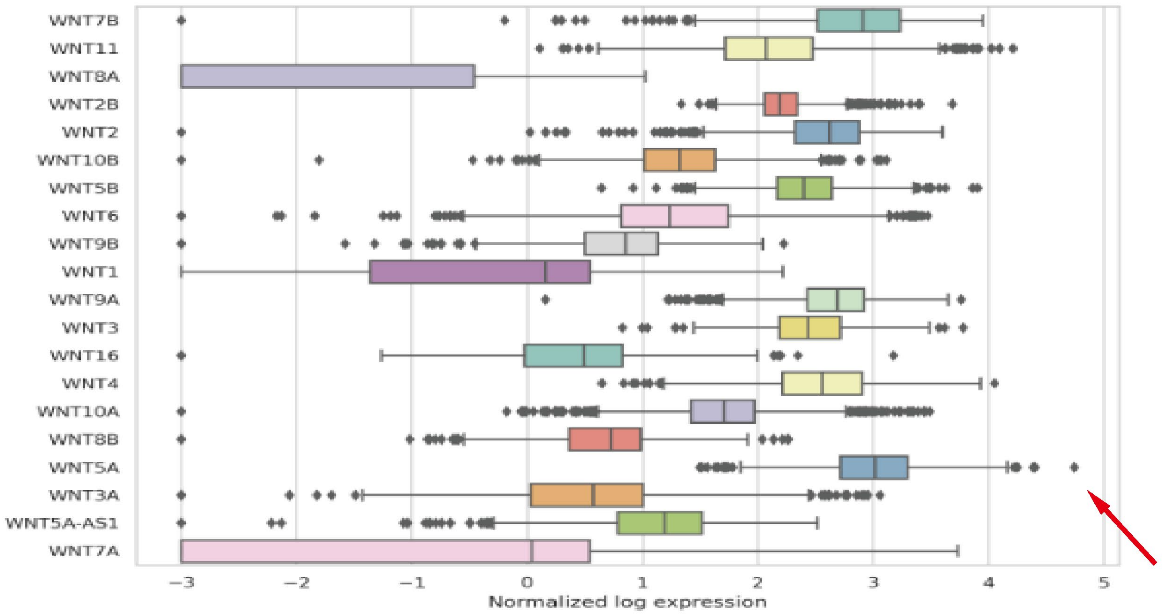

Supplemental Figure 8 Expression of WNT genes in the inhouse scRNA-seq dataset.

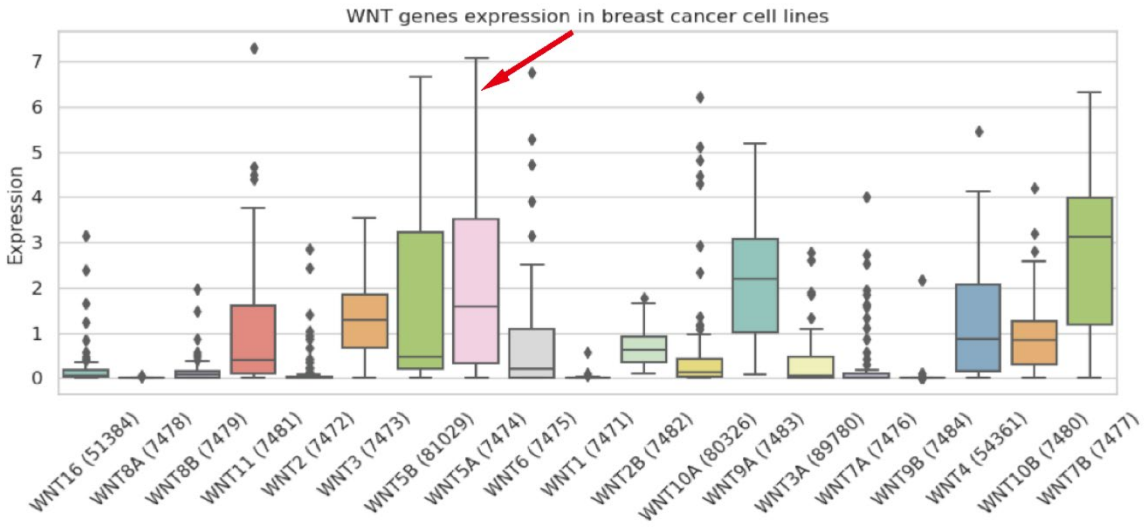

Supplemental Figure 9 Expression of WNT genes in CCLE dataset.

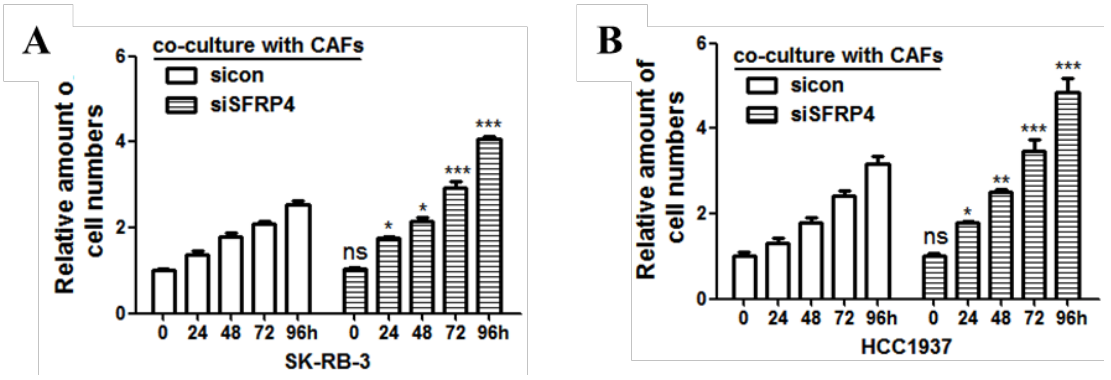

Supplemental Figure 10 Cell proliferation rate of SK-BR-3 and HCC1937 cells when co-culture with siSFRP4-CAFs (A) SK-RB-3 (B) HCC1937

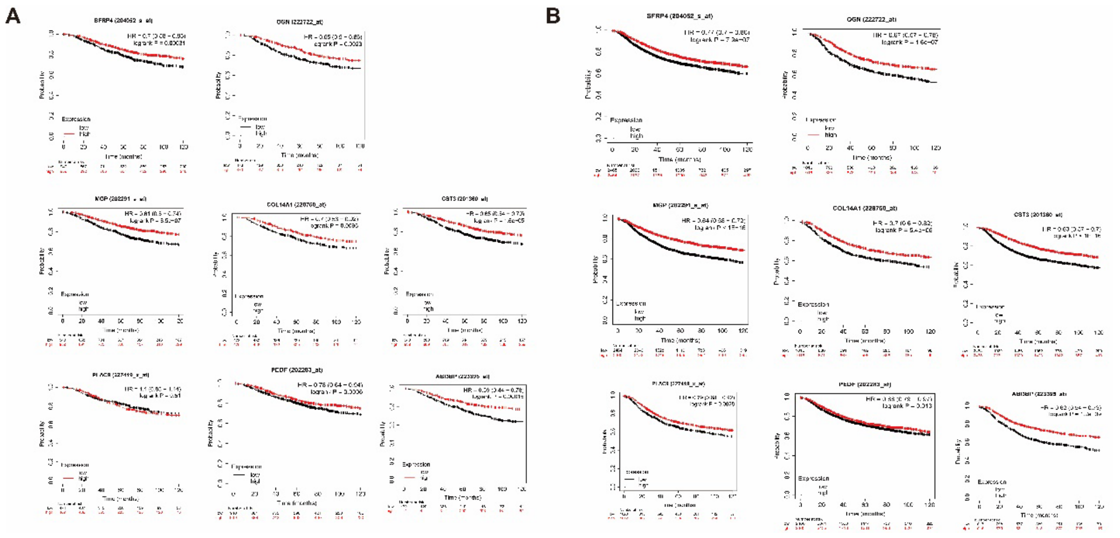

Supplemental Figure 11 Kaplan–Meier curve of SFRP4+ CAF marker genes from Kaplan-Meier Plotter. (A) Overall survival (B) Relapse-free survival
